# Author Correction: 53BP1 regulates heterochromatin through liquid phase separation

**DOI:** 10.1038/s41467-022-28780-0

**Published:** 2022-02-23

**Authors:** Lei Zhang, Xinran Geng, Fangfang Wang, Jinshan Tang, Yu Ichida, Arishya Sharma, Sora Jin, Mingyue Chen, Mingliang Tang, Franklin Mayca Pozo, Wenxiu Wang, Janet Wang, Michal Wozniak, Xiaoxia Guo, Masaru Miyagi, Fulai Jin, Yongjie Xu, Xinsheng Yao, Youwei Zhang

**Affiliations:** 1 Department of Pharmacology, Case Comprehensive Cancer Center, Case Western Reserve University, School of Medicine, Cleveland, OH 44106 USA; 2 Institute of Traditional Chinese Medicine and Natural Products, College of Pharmacy, Jinan University, Guangzhou, 510632 China; 3 College of Life Sciences, Wuhan University, Wuhan, Hubei 430068 China; 4 Department of Genetics and Genome Sciences, Case Western Reserve University, School of Medicine, Cleveland, OH 44106 USA; 5 Department of Pharmacology and Toxicology, Wright State University, Dayton, OH 45435 USA; 6 Present Address: National 111 Center for Cellular Regulation and Molecular Pharmaceutics, Key Laboratory of Fermentation Engineering, Hubei University of Technology, Wuhan, Hubei 430068 China; 7 Present Address: Department of Molecular Biology of Cancer, Medical University of Lodz, 6/8 Mazowiecka Street, 92-215 Lodz, Poland

**Keywords:** Cell biology, Molecular biology

Correction to: *Nature Communications* 10.1038/s41467-022-28019-y, published online 18 January 2022.

The original version of this Article incorrectly acknowledged Zhunkun Lou instead of Zhenkun Lou in the Acknowledgements section. This has now been corrected in both the PDF and HTML versions of the Article.

