# Supplementary material for: 53BP1 regulates heterochromatin through liquid phase separation
Source: Nat Commun. 2022 Jan 18;13:360. doi: 10.1038/s41467-022-28019-y (PMC8766474; doi:10.1038/s41467-022-28019-y)

53BP1 regulates heterochromatin through liquid phase separation

Lei Zhang[1,2,4*], Xinran Geng[1,4], Fangfang Wang[3,4], Jinshan Tang[3], Yu Ichida[1], Arishya Sharma[1], Sora Jin[1], Mingyue Chen[2], Mingliang Tang[5], Franklin Mayca Pozo[1], Wenxiu Wang[2], Janet Wang[6], Michal Wozniak[7,†], Xiaoxia Guo[2], Masaru Miyagi[1], Fulai Jin[6], Yongjie Xu[7], Xinsheng Yao[3], and Youwei Zhang[1,*]

[1] Department of Pharmacology, Case Comprehensive Cancer Center, Case Western Reserve University, School of Medicine, Cleveland, OH 44106, USA.

[2] Current address: National 111 Center for Cellular Regulation and Molecular Pharmaceutics, Key Laboratory of Fermentation Engineering, Hubei University of Technology, Wuhan, Hubei 430068, China.

[3] Institute of Traditional Chinese Medicine and Natural Products, College of Pharmacy, Jinan University, Guangzhou 510632, China.

[5] College of Life Sciences, Wuhan University, Wuhan, Hubei 430068, China.

[6] Department of Genetics and Genome Sciences, Case Western Reserve University, School of Medicine, Cleveland, OH 44106, USA.

[7] Department of Pharmacology and Toxicology, Wright State University, Dayton, OH 45435, USA.

[†] Current address: Department of Molecular Biology of Cancer, Medical University of Lodz, 6/8 Mazowiecka Street, 92-215 Lodz, Poland.

[4] These authors contributed equally: Lei Zhan, Xinran Geng, Fangfang Wang

[*] These authors jointly supervised this work: Lei Zhang, Youwei Zhang

zhanglei0222@163.com
yxz169@case.edu

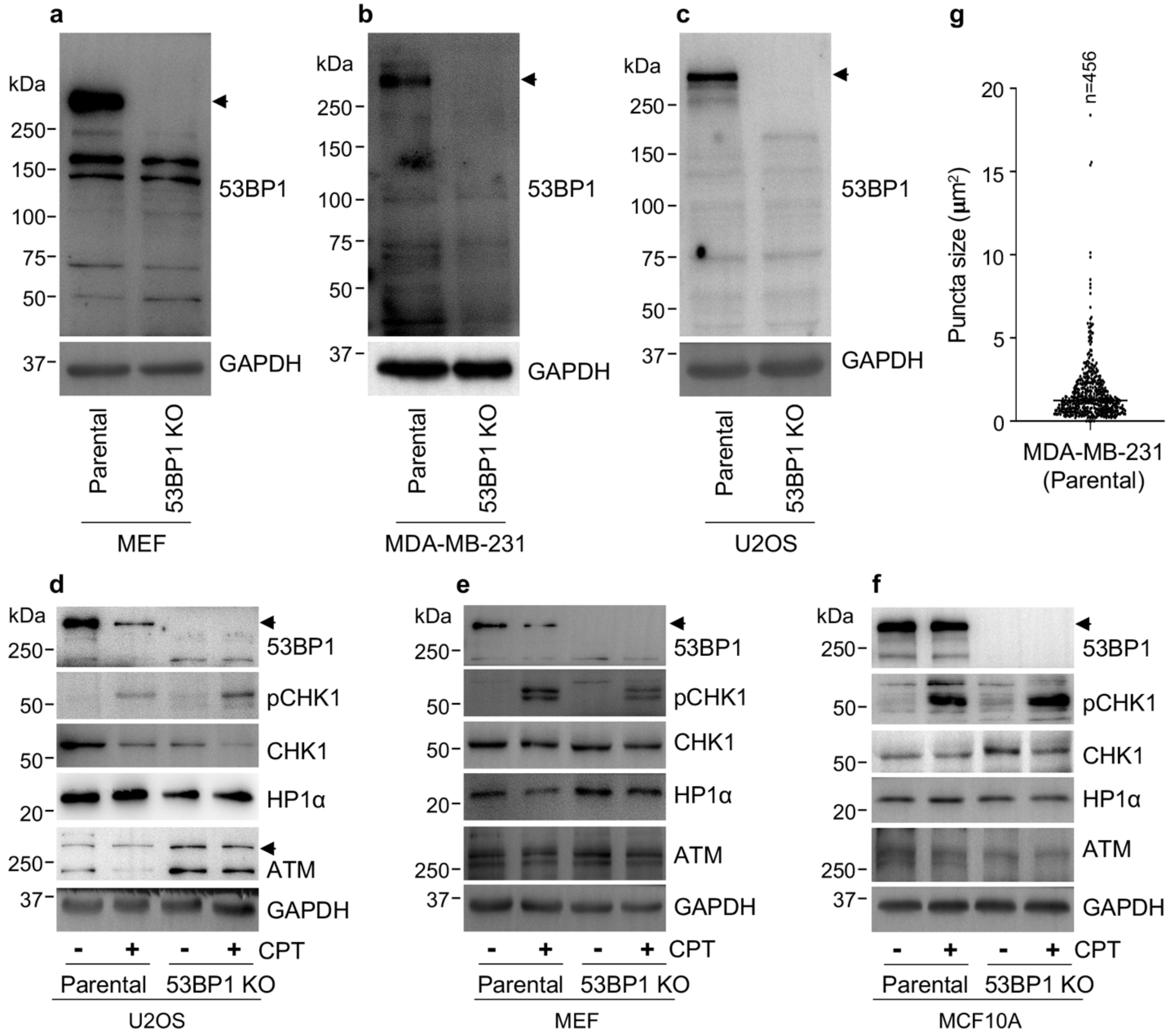

Supplementary Fig. 1. Validation of 53BP1 knockout (KO) cell lines and 53BP1 puncta size distribution. Equal amount of proteins from parental and 53BP1 KO MEF (**a**), MDA-MB-231 (**b**) or U-2 OS (**c**) cells were run on 6-12% SDS-PAGE and blotted with anti-53BP1 and anti-GAPDH antibodies. Arrows indicate the band corresponding to full length 53BP1. Note additional bands were detected, which were also seen in overexpressed GFP-tagged 53BP1 proteins (see below), indicating transcriptional variants or protein truncation of 53BP1, which needs further investigation. Parental and 53BP1 KO U-2 OS (**d**), MEF (**e**) or MCF10A (**f**) cells were treated with 500 nM CPT for 6 h and protein expression was examined. **g**, Size distribution of 53BP1 puncta in MDA-MB-231 cells under normal growth conditions. A total n=456 puncta was analyzed by the NIH Image J software. One sample t and Wilcoxon test was done by Prism 9.0 for statistical analysis. The median size of 53BP1 punctum was 1.243 μm^2 (in area). The 5%, 25%, 75% and 95% percentiles were 0.295, 0.680, 2.196 and 5.178 μm^2, respectively.

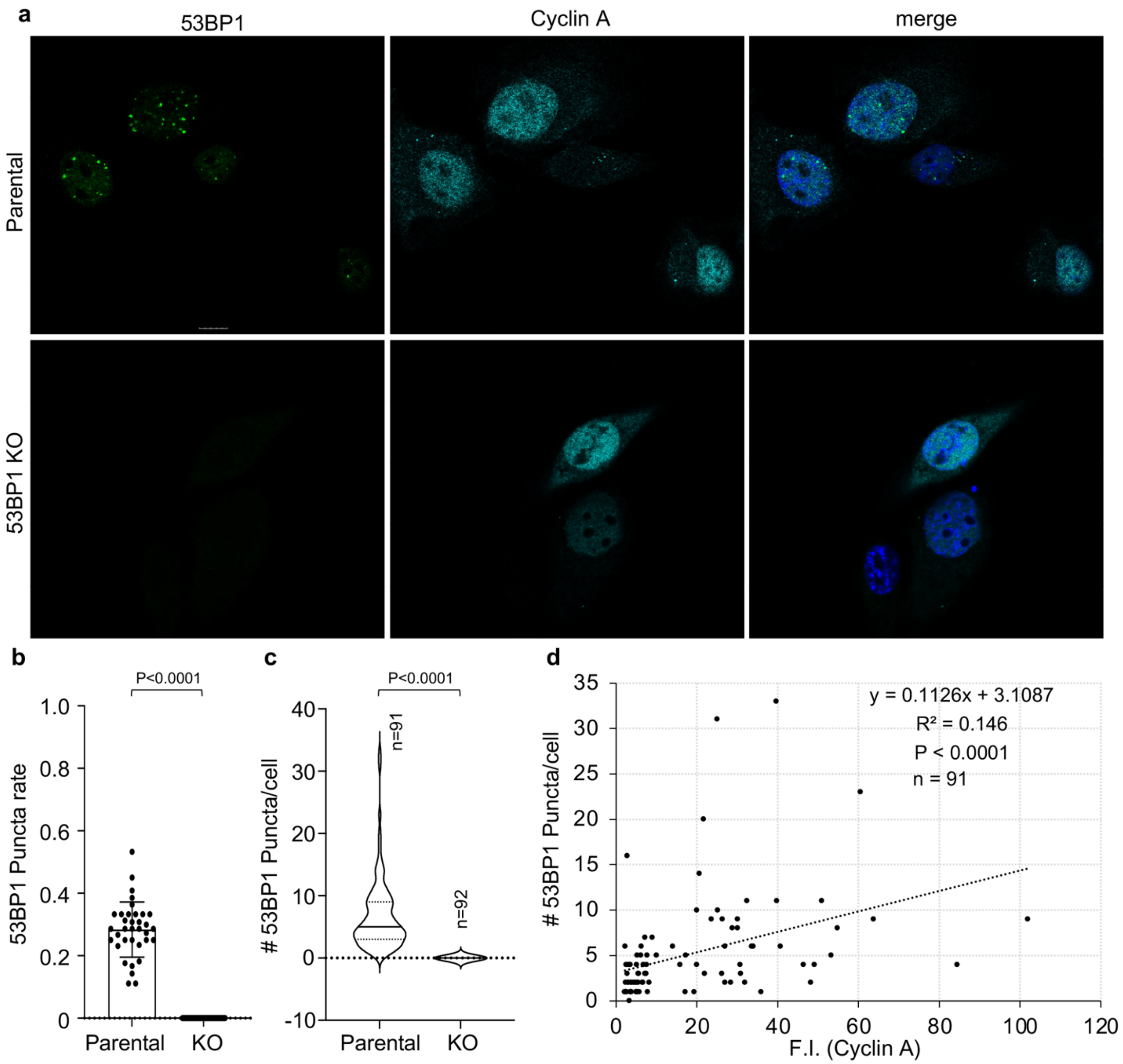

Supplementary Fig. 2. Cell cycle dependency of 53BP1 puncta in U-2 OS cells. **a**, Representative single z-plane confocal images of endogenous 53BP1 and Cyclin A in U-2 OS parental and 53BP1 KO cells under normal growth conditions. Scale bar is 10 μm. **b**, Percentage of 53BP1 puncta rate was analyzed from n=36 and 35 images acquired from three independent experiments for parental and KO groups with a total number of 425 and 186 cells, respectively. Data represent mean values and standard deviation (SD). **c**, Violin plot of 53BP1 puncta number per cell from 91 and 92 parental and 53BP1 KO U-2 OS cells, respectively, done in two independent experiments. Data represent mean, 25th and 75th percentiles with the whiskers extending to the minimum and maximum values. **d**, Correlation of 53BP1 puncta number and Cyclin A expression levels (expressed as mean fluorescence intensity, F.I.) was analyzed from indicated number (n) of parental U-2 OS cells obtained from duplicate experiments. Each dot represents one cell. Unpaired two-tailed t test using Prism 9.0 was conducted for ***b*** and ***c*** with 95% confidence intervals, whereas the P-Value in ***d*** was acquired by the Pearson Correlation Coefficient Calculator.

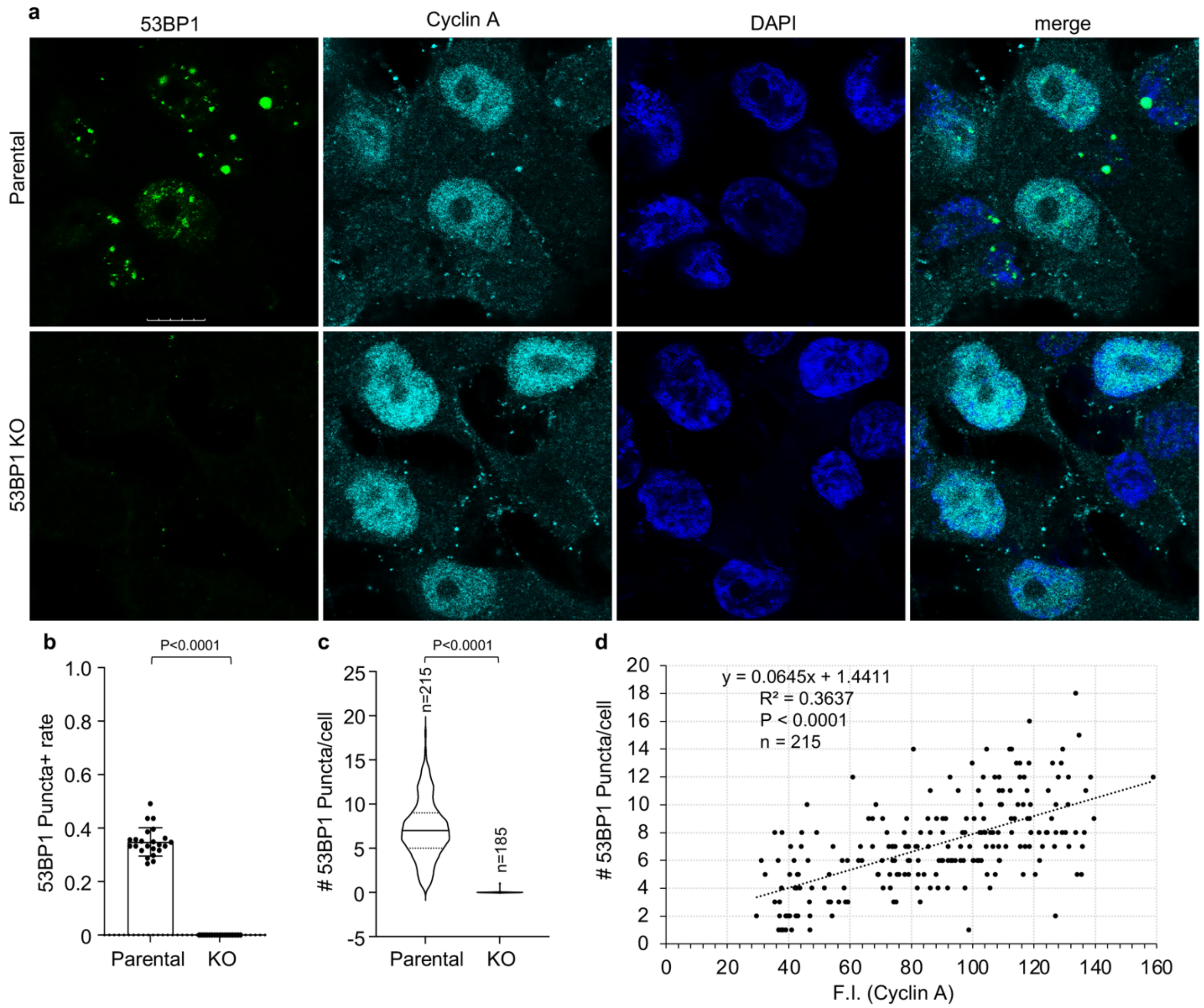

Supplementary Fig. 3. Cell cycle dependency of 53BP1 nuclear puncta in MCF10A cells. **a**, Representative single z-plane confocal images of endogenous 53BP1 and Cyclin A in MCF10A parental and 53BP1 KO cells under normal growth conditions. Scale bar is 10 µm. **b**, Percentage of 53BP1 puncta rate was analyzed from n=23 images acquired from three independent experiments containing a total number of 1399 and 720 cells for parental and 53BP1 KO groups, respectively. Data represent mean values and SD. **c**, Violin plot of 53BP1 puncta number per cell was analyzed from n=215 and 185 parental and KO MCF10A cells, respectively, acquired from two independent experiments. Data represent mean, 25th and 75th percentiles with the whiskers extending to the minimum and maximum values. **d**, Correlation of 53BP1 puncta number and Cyclin A expression levels was analyzed from n=215 MCF10A parental cells from duplicate experiments. Each dot represents one cell. Unpaired two-tailed t test using Prism 9.0 was conducted for ***b*** and ***c*** with 95% confidence intervals, whereas the P-Value in ***d*** was acquired by the Pearson Correlation Coefficient Calculator.

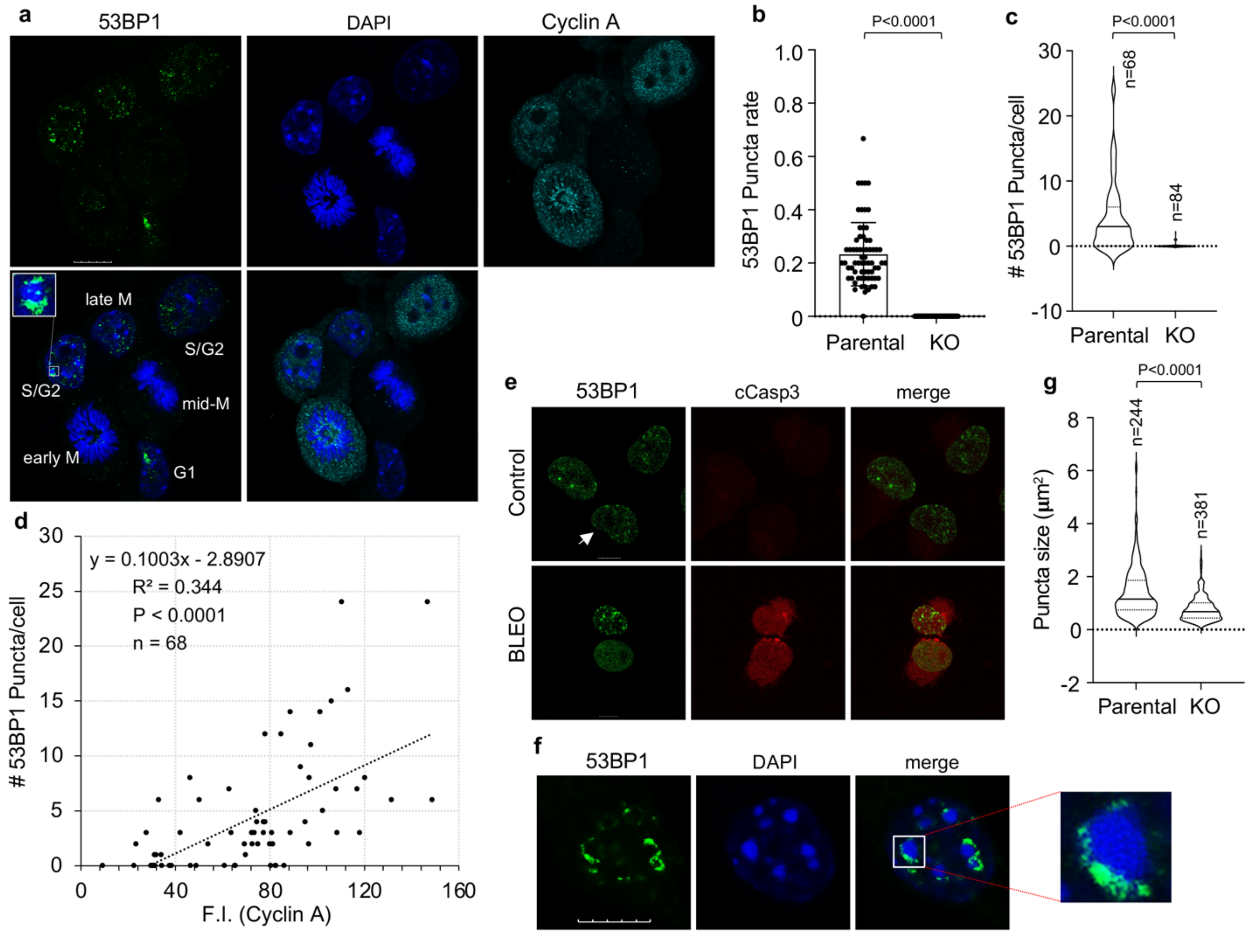

Supplementary Fig. 4. Regulation of 53BP1 nuclear puncta. **a**, Representative single z-plane confocal images of endogenous 53BP1 and Cyclin A in MEF parental cells under normal growth conditions. Cell cycle was identified by Cyclin A and DAPI-determined DNA staining pattern. Scale bar is 10 µm. *Square*: an enlarged area. **b**, Percentage of 53BP1 puncta rate was analyzed from n=67 and 79 images acquired from three independent experiments with a total number of 456 and 433 cells for parental and KO groups, respectively. Data represent mean values and SD. **c**, Violin plot of 53BP1 puncta number per cell was analyzed from n=68 and 84 parental and KO MEF cells, respectively, obtained from two indepedent experiments. Data represent mean, 25th and 75th percentiles with the whiskers extending to the minimum and maximum values. **d**, Correlation of 53BP1 puncta number and Cyclin A expression levels was analyzed from n=68 parental MEF cells. Each dot represents one cell. **e**, U-2OS parental cells were treated or not with 3.5 µM bleomycin (BLEO) for 6 h, fixed and stained with antibodies against endogenous 53BP1 and cleaved Caspase 3 (cCasp3). The arrow indicates a cell with less circular nuclear shape. **f**, Representative single z-plane confocal images showing the co-localization of 53BP1 puncta at DAPI-coated heterochromatin centers in parental MEFs. Scale bar is 10 µm. *Square*: an enlarged heterochromatin center. **g**, H3K9me3 puncta size in MDA-MB-231 cells was measured by the Image J software and presented as Violin plot from n=224 and 381 puncta in parental and KO cells, respectively. Data represent mean, 25th and 75th percentiles with the whiskers extending to the minimum and maximum values. Unpaired two-tailed t test using Prism 9.0 was performed for ***b***, ***c*** and ***g*** with 95% confidence intervals, whereas the P-Value in ***d*** was performed by Pearson Correlation Coefficient Calculator.

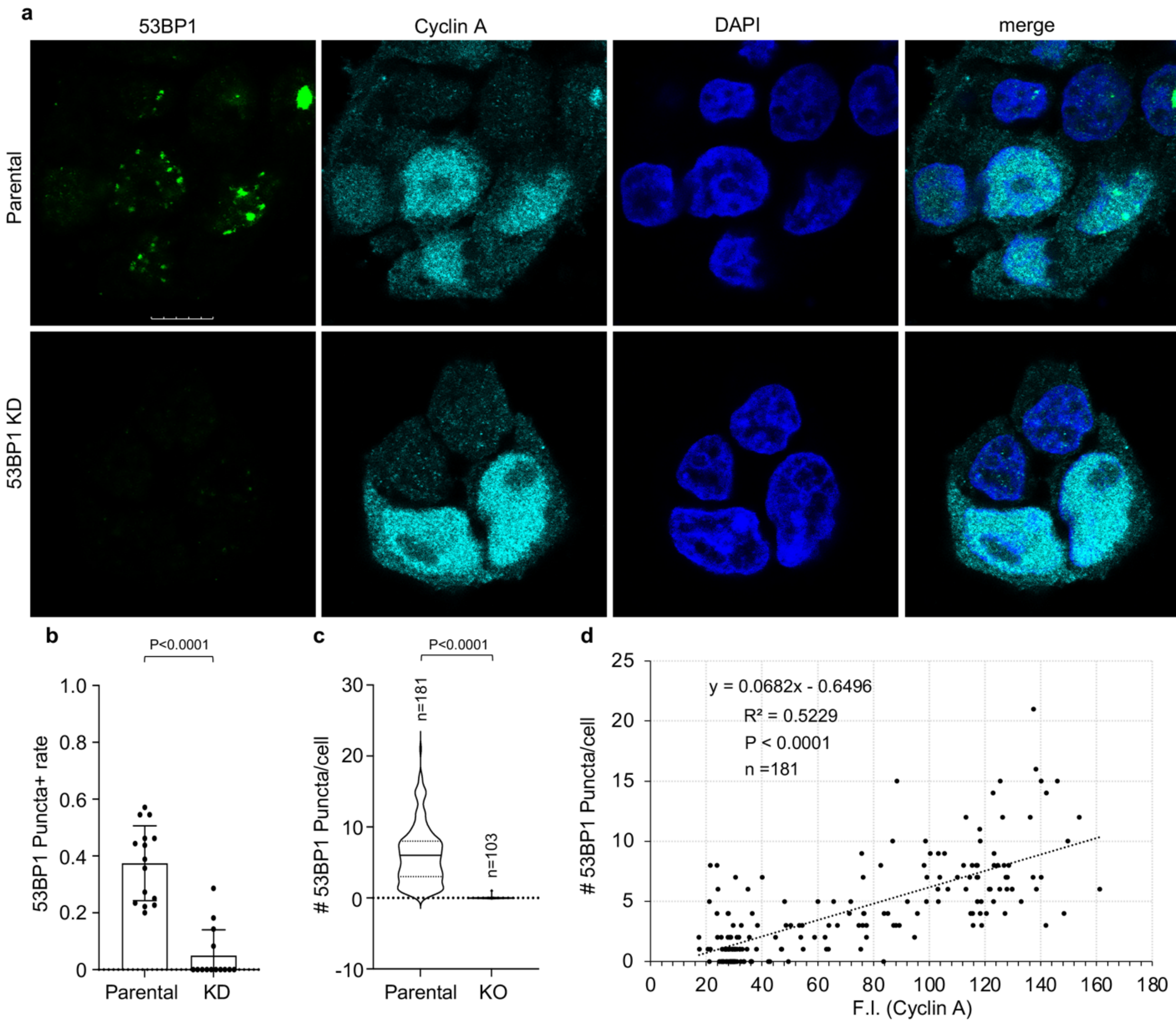

Supplementary Fig. 5. Cell cycle dependency of 53BP1 nuclear puncta in HEK293T cells. **a**, Representative single z-plane confocal images of endogenous 53BP1 and Cyclin A in HEK293T parental and 53BP1 KD cells under normal growth conditions. Scale bar is 10 µm. **b**, Percentage of 53BP1 puncta rate was analyzed from n=15 and 14 images acquired from two independent experiments with a total number of n=206 and 74 parental and KD cells, respectively. Data represent mean values and SD. **c**, Violin plot of 53BP1 puncta number per cell was analyzed from n=181 and 103 parental and 53BP1 KD HEK293T cells, respectively, acquired from two independent experiments. Data represent mean, 25th and 75th percentiles with the whiskers extending to the minimum and maximum values. **d**, Correlation of 53BP1 puncta number and Cyclin A expression levels was analyzed from n=181 HEK293T parental cells. Each dot represents one cell. Unpaired two-tailed t test using Prism 9.0 was performed in ***b*** and ***c*** with 95% confidence intervals, whereas the P-Value in ***d*** was from the Pearson Correlation Coefficient Calculator.

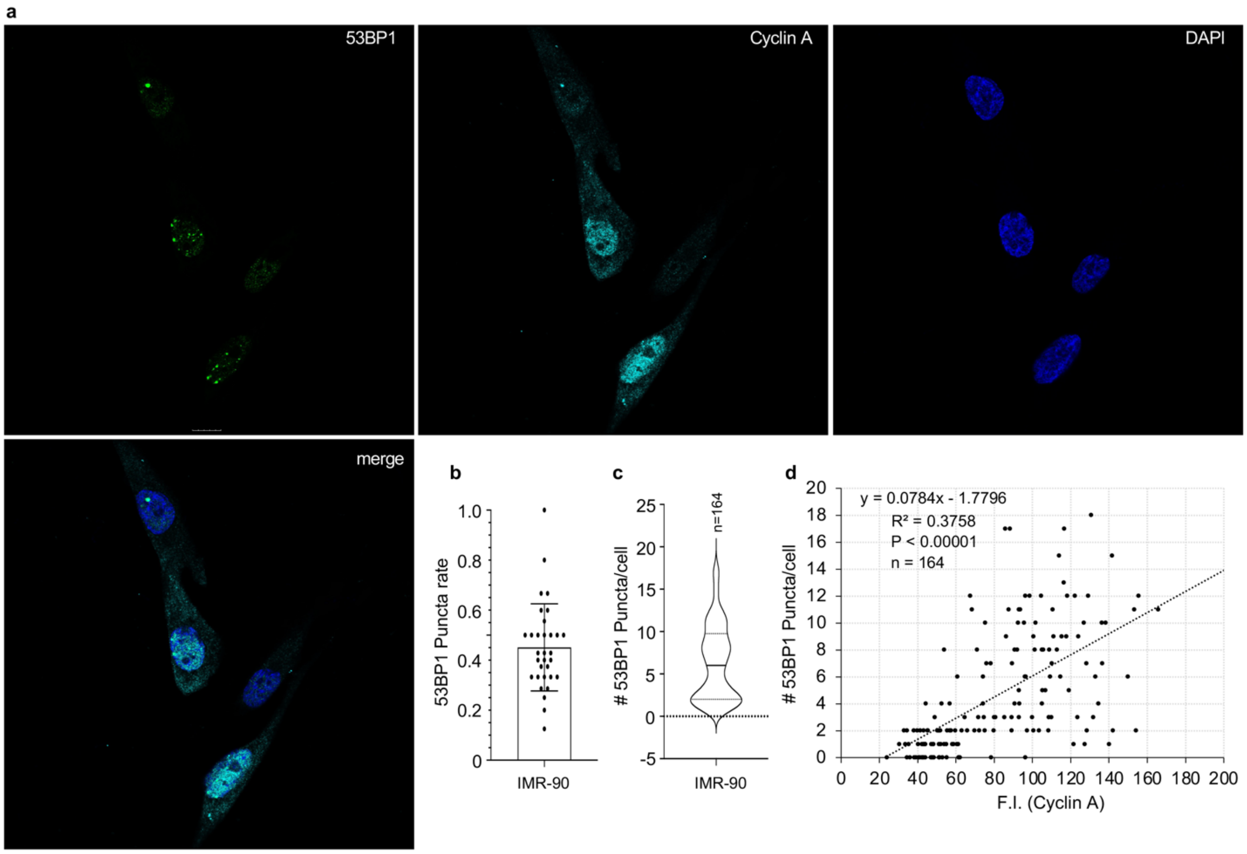

Supplementary Fig. 6. 53BP1 puncta formation in IMR90 cells. **a**, Representative single z-plane confocal images of endogenous 53BP1 and Cyclin A in IMR-90 cells under normal growth conditions. Scale bar is 10 µm. **b**, Percentage of 53BP1 puncta rate was analyzed from n=32 images with a total number of 164 cells obtained from two independent experiments. Data represent mean value and SD. **c**, Violin plot of 53BP1 puncta number per cell was analyzed from n=164 cells from two independent experiments. Data represent mean, 25th and 75th percentiles with the whiskers extending to the minimum and maximum values. **d**, Correlation of 53BP1 puncta number and Cyclin A expression levels was analyzed from n=164 cells. Each dot represents one cell. The P-Value is <0.00001 by Pearson Correlation Coefficient Calculator.

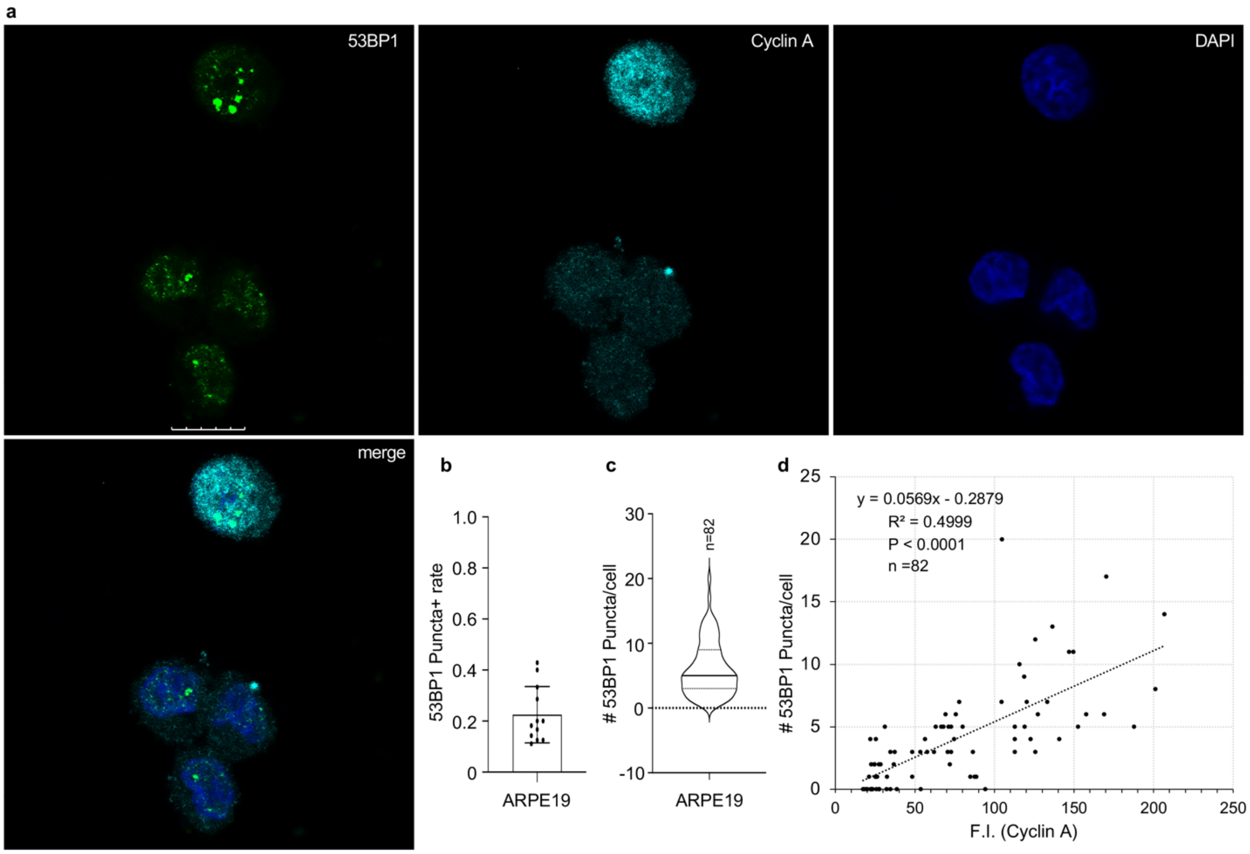

Supplementary Fig. 7. 53BP1 puncta in ARPE19 cells. **a**, Representative single z-plane confocal images of endogenous 53BP1 and Cyclin A in ARPE19 cells under normal growth conditions. Scale bar is 10 µm. **b**, Percentage of 53BP1 puncta rate was analyzed from n=12 images with a total number of 82 cells from two independent experiments. Data represent mean value and SD. **c**, Violin plot of 53BP1 puncta number per cell was analyzed from n=82 cells acquired from two independent experiments. Data represent mean, 25th and 75th percentiles with the whiskers extending to the minimum and maximum values. **d**, Correlation of 53BP1 puncta number and Cyclin A expression levels was analyzed from n=82 cells. Each dot represents one cell. The P-Value is <0.00001 by the Pearson Correlation Coefficient Calculator.

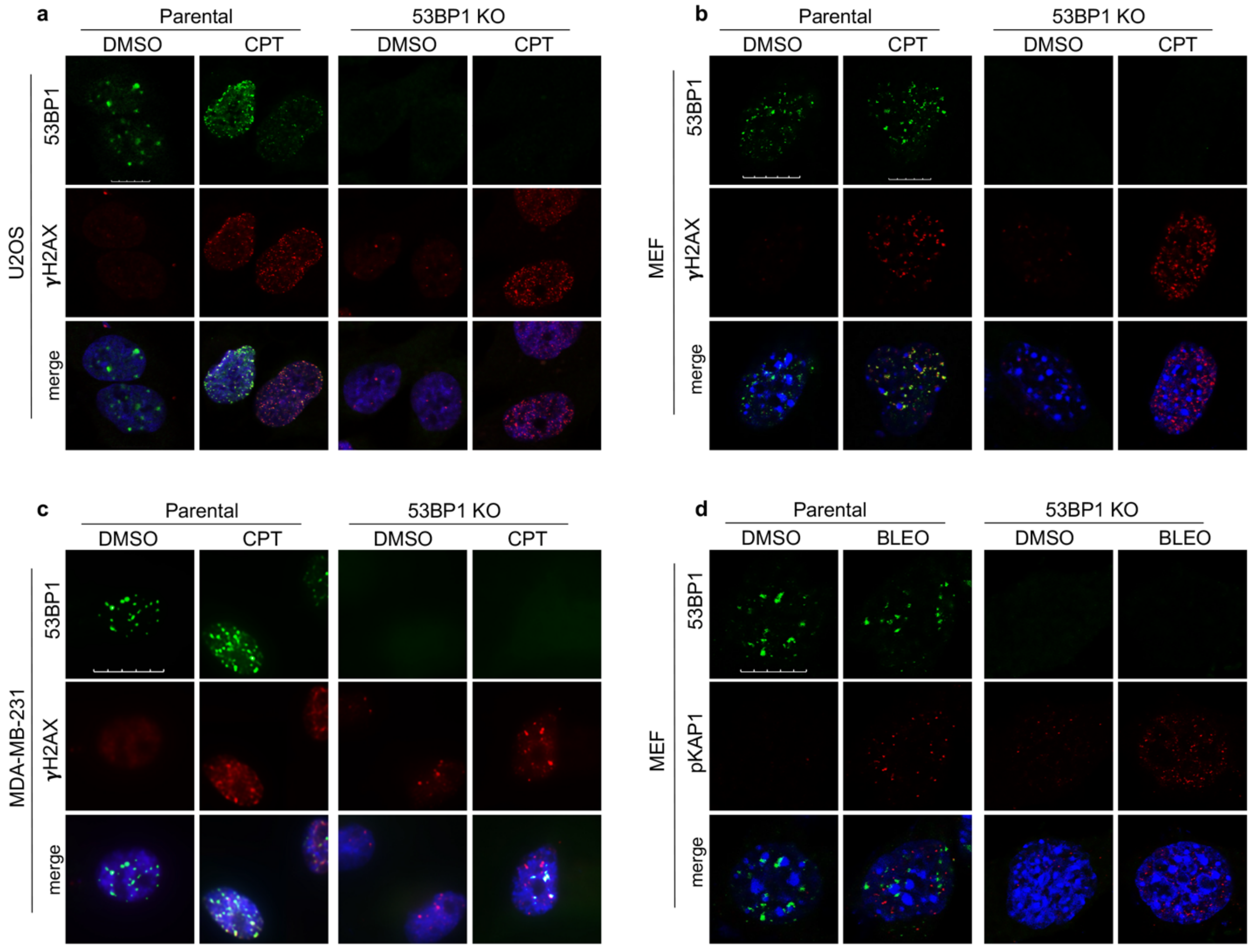

Supplementary Fig. 8. 53BP1 puncta did not represent DNA damage. Representative images of endogenous 53BP1 and γH2AX in parental and 53BP1 KO U-2 OS (**a**), MEF (**b**) or MDA-MD-231 (**c**) cells treated or not with 500 nM CPT for 6 h. **d**, Representative images of endogenous 53BP1 and pKAP1 in parental and 53BP1 KO MEFs treated or not with 3.5 µM BLEO for 6 h. Except projection images are shown in ***c***, single z-plane confocal images are shown for ***a***, ***b*** and ***d***. Scale bar in all images is 10 µm.

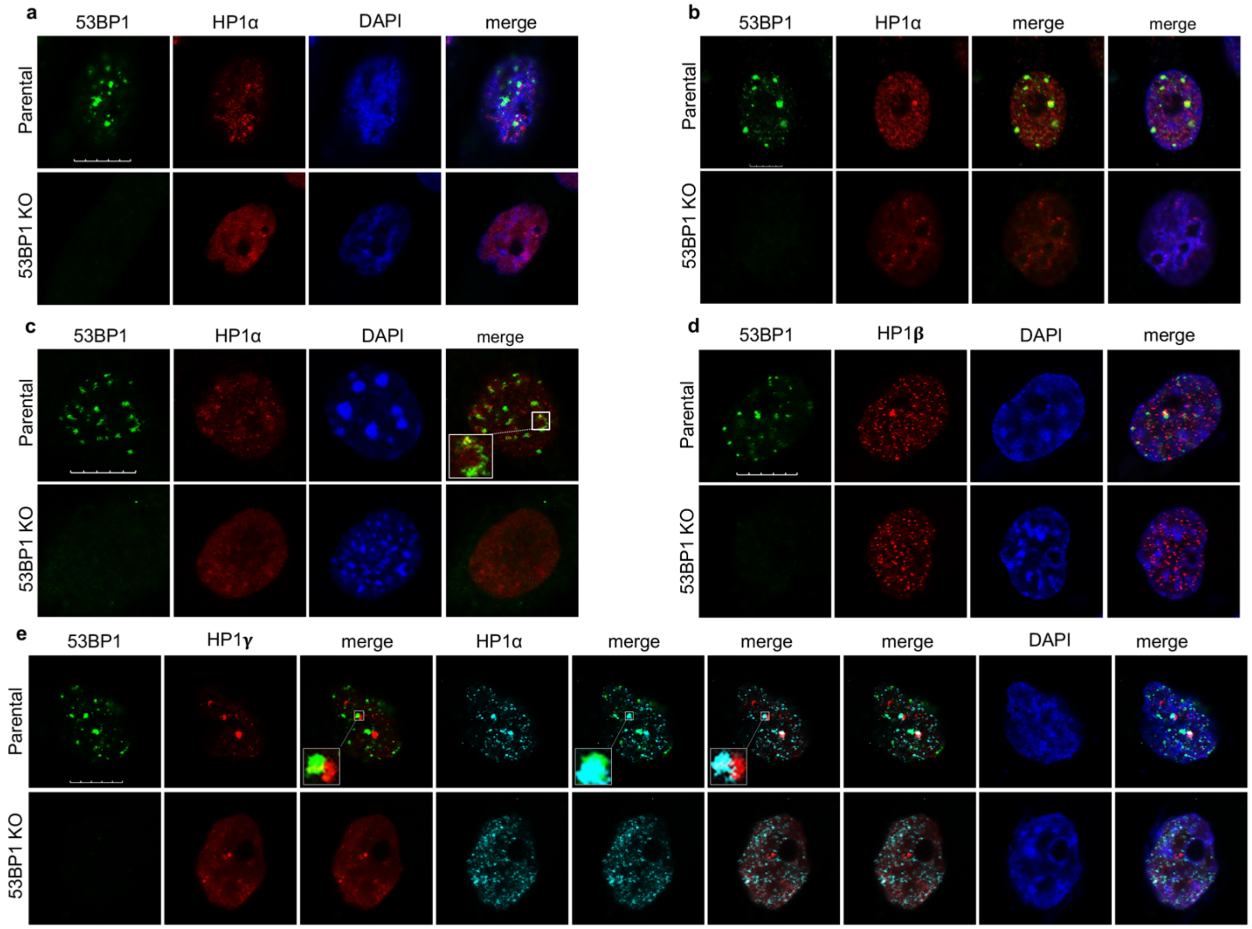

Supplementary Fig. 9. 53BP1 puncta co-localize with heterochromatin. Representative single z-plane confocal images of endogenous 53BP1 and HP1α in parental and 53BP1 KO MDA-MB-231 (**a**), U-2 OS (**b**) or MEF (**c**) cells or HP1β in MDA-MB-231 cells (**d**). **e**, Representative single z-plane confocal images of endogenous 53BP1, HP1α and HP1γ in parental and 53BP1 KO MDA-MB-231 cells. Scale bar is 10 µm. *Squares*: enlarged areas.

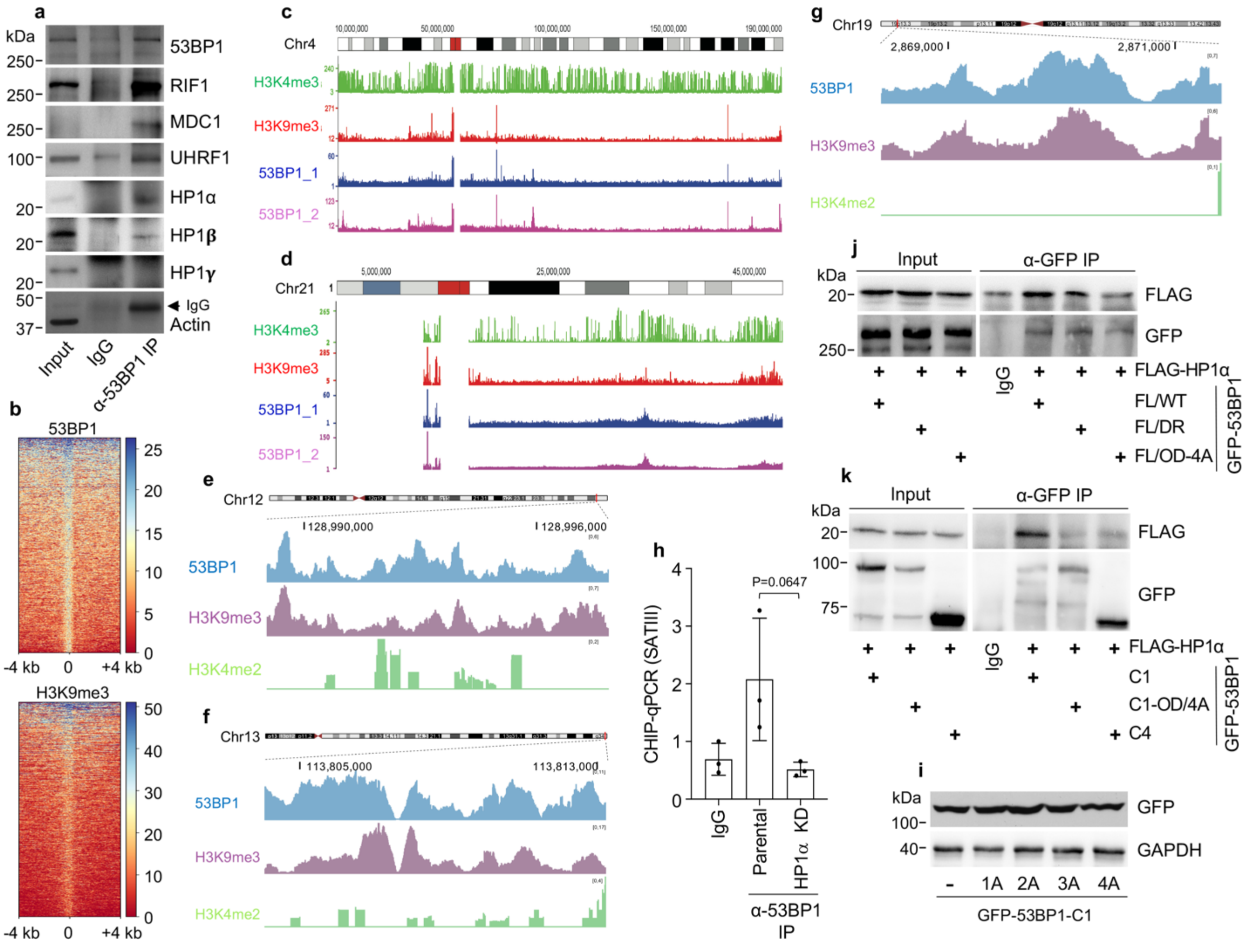

Supplementary Fig. 10. Heterochromatin localization of 53BP1. **a**, Parental U-2 OS cells were IPed with IgG or anti-53BP1 (mouse, Sigma) and blotted with indicated antibodies (including rabbit anti-53BP1 from Novus). **b**, Heat maps showing whole genome-wide ChIP–seq enrichment (log2[ChIP/input]) of 53BP1 and H3K9me3 on 53BP1 peaks in U2OS cells [40]. Peaks of 53BP1 and H3K9me3 intersected with γH2AX were filtered to avoid the effect of DNA double strand breaks. Distance from center of peak in base pairs is shown on x-axis. Enrichments are ranked according to 53BP1 enrichment in descending order. **c-d**, ChIP-seq data from U-2 OS cells were obtained from the publicly available E-MTAB-5817 from EMBL-EBL ArrayExpress database and SRR10540101 from SRA [40, 41]. Data were downloaded in FASTQ format and aligned to human genome hg38. The aligned files were converted to bigwig files and uploaded to the Genome Browser for visual analysis. Two 53BP1 biological replicates were compared to that of H3K9me3 or H3K4me3 on chromosomes 4 (**c**) and 21 (**d**). **e-g**, Focused areas of 53BP1, H3K9me3 and H3K4me2 on representative chromosomes. **h**, ChIP-qPCR of 53BP1 in parental and HP1α depleted U-2 OS cells. Data represent mean values and SD from n=3 biological replicates. Unpaired two-tailed t-test was conducted for statistical analysis with 95% confidence intervals. **i**, Protein expression of GFP-53BP1-C1 mutants. U-2 OS cells were transfected with GFP-53BP1-C1 or OD mutants (1A to 4A indicating Ala mutations of the four conserved residues in the OD region) for 48 h, and protein expression was assessed. **j**, HEK293T cells were transfected with FLAG-HP1α and GFP-53BP1-FL/WT, -FL/DR or -FL/OD-4A for 48 h, lysed and IPed with anti-GFP, and blotted with indicated antibodies. Protein levels in the input were also examined. **k**, HEK293T cells were transfected with FLAG-HP1α and GFP-53BP1-C1, -C1/OD-4A or -C4 for 48 h, lysed and IPed with anti-GFP, and blotted with indicated antibodies.

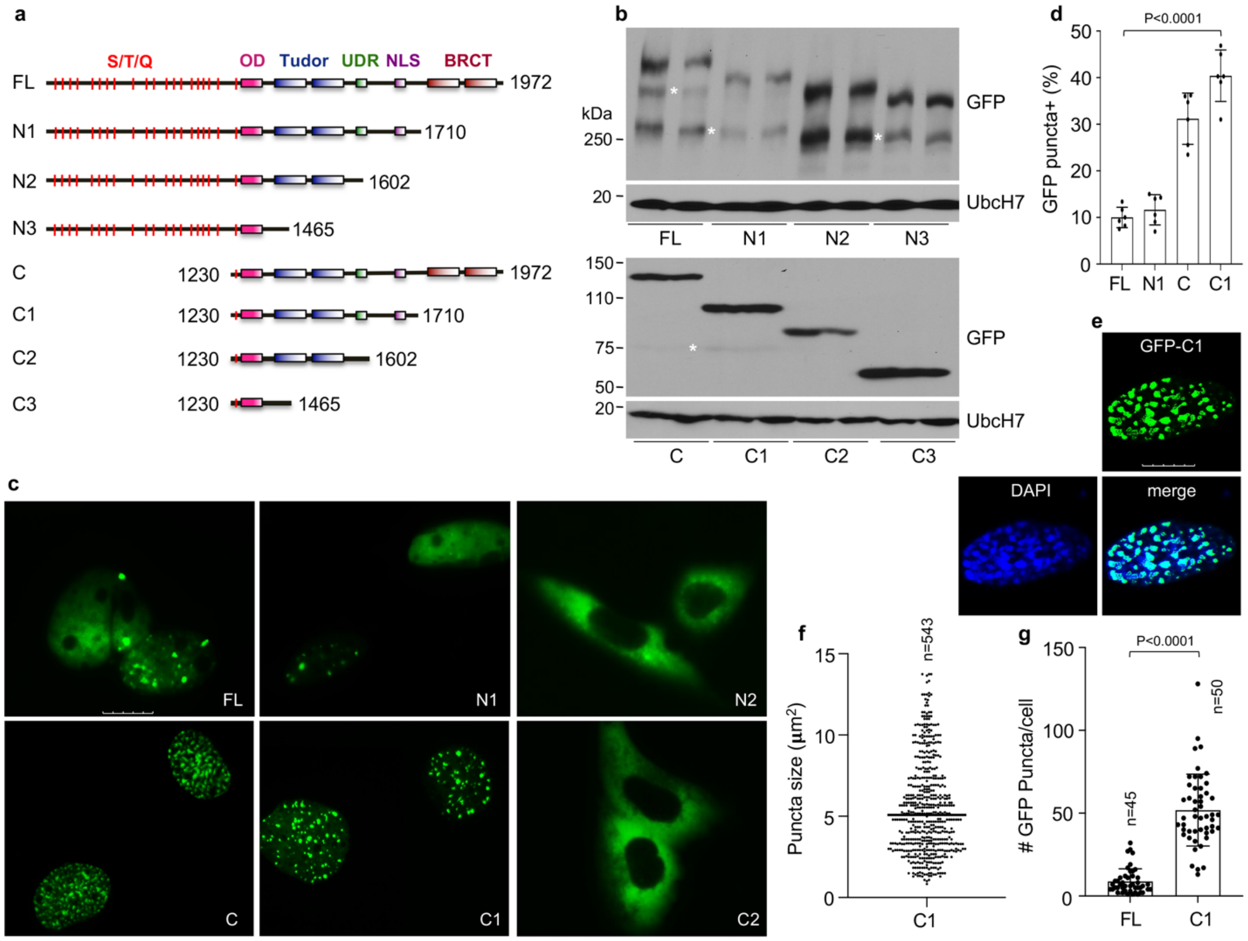

Supplementary Fig. 11. Mapping domains of 53BP1 for puncta formation. **a**, Schematic diagram for generating GFP-tagged 53BP1 constructs. **b**, U-2 OS cells were transfected with GFP-53BP1 constructs for 48 h and protein expression was examined. White asterisk markers indicate either transcript variants or truncation of 53BP1 as seen in endogenous proteins (Supplementary Fig. 1a-c). **c**, Representative projection images of GFP-53BP1 proteins expressed in U-2 OS cells. Scale bar is 16 μm. **d**, Quantitation of GFP puncta positive cells in ***c*** from n=6 replicates from two independent experiments. Data represent mean values and SD. **e**, U-2 OS cells were transfected with GFP-53BP1-C1 for 48 h, fixed and stained with DAPI. Representative single z-plane confocal images are shown for the 53BP1-C1 puncta/DAPI co-localization. Scale bar is 10 μm. **f**, Size distribution of GFP-53BP1-C1 puncta in U-2 OS cells. A total n=543 puncta was analyzed by the NIH Image J software from three independent experiments. One sample t and Wilcoxon test was done by Prism 9.0 for statistical analysis. The median size of GFP-53BP1-C1 puncta was estimated to be 5.081 μm^2 (in area). The 25%, 75% and 95% percentiles were 3.290, 7.330 and 11.130 μm^2, respectively. **g**, Number of GFP puncta per cell formed by GFP-53BP1-FL or GFP-53BP1-C1 from indicated number of cells. Data represent mean values and SD. Unpaired two-tailed t test using Prism 9.0 was analyzed for statistical significance with 95% confidence intervals.

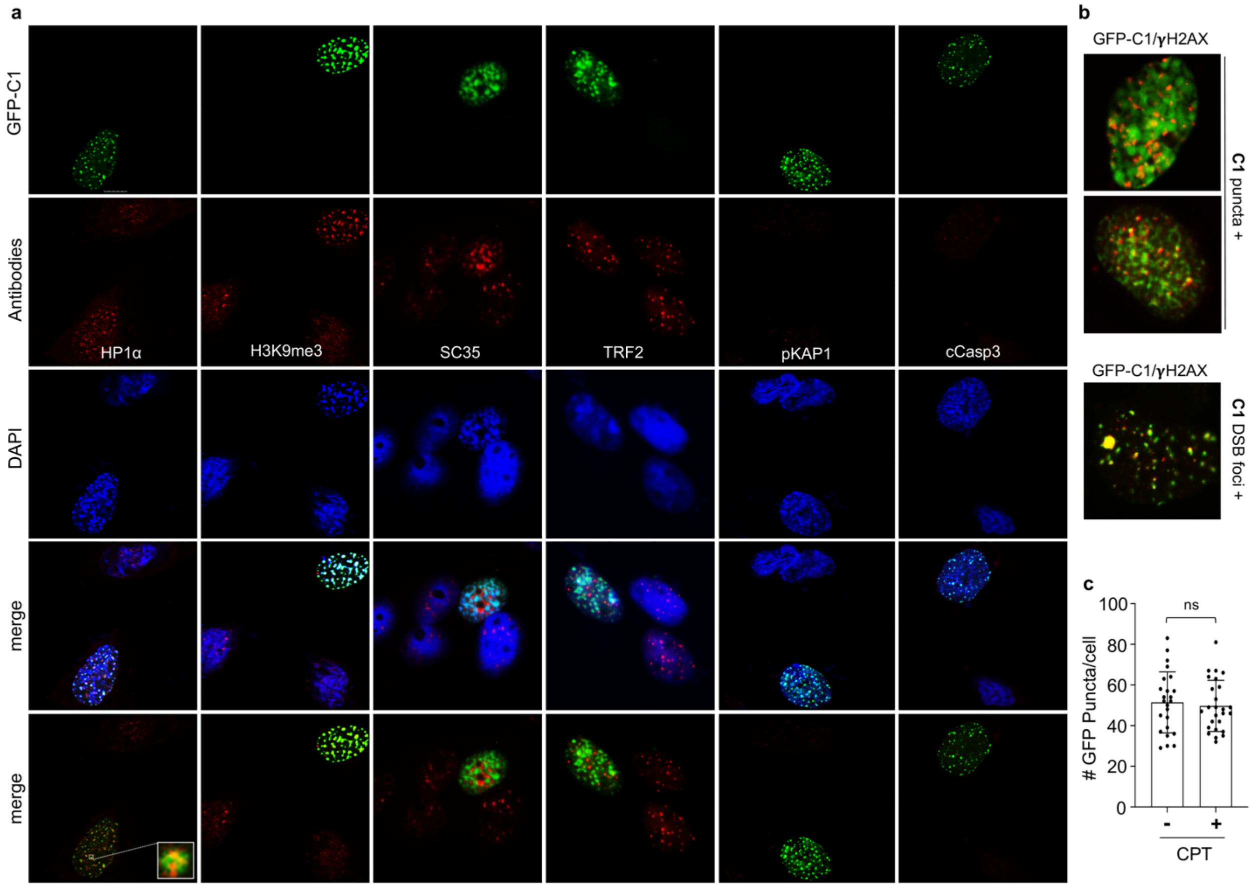

Supplementary Fig. 12. GFP-53BP1-C1 puncta localize at heterochromatin. **a**, U-2 OS cells were transfected with GFP-53BP1-C1 for 48 h, fixed and stained with indicated antibodies. Representative images for each antibody group are shown. Scale bar is 10 µm. *Square*: an enlarged area. **b**, U-2 OS cells were transfected with GFP-53BP1-C1 for 48 h, treated with 500 nM CPT for 6 h, fixed and stained with antibodies against γH2AX. Generally, two types of 53BP1-C1 'dots' were detected. In one type, the 'dots' rarely co-localized with γH2AX foci (*upper*); even if they co-localized, it was a limited level of partial co-localization. These 'dots' were determined to be DSB-repair independent puncta. In the other type, the 53BP1-C1 'dots' displayed a high level of co-localization with γH2AX foci (*lower*). This type of 53BP1-C1 'dots' was counted as DSB foci. **c**, U-2 OS cells were transfected with GFP-53BP1-C1 for 48 h, treated with 500 nM CPT for 6 h, fixed and stained with anti-γH2AX. The number of GFP puncta per cell that had limited co-localization with γH2AX (*i.e.*, those in the upper panel in Supplementary Fig. 12b) were counted from n=24 and 26 images for DMSO and CPT, respectively, and presented as mean values and SD from two independent experiments. ns: not significant from unpaired two-tailed t-test by Prism 9 with 95% confidence intervals.

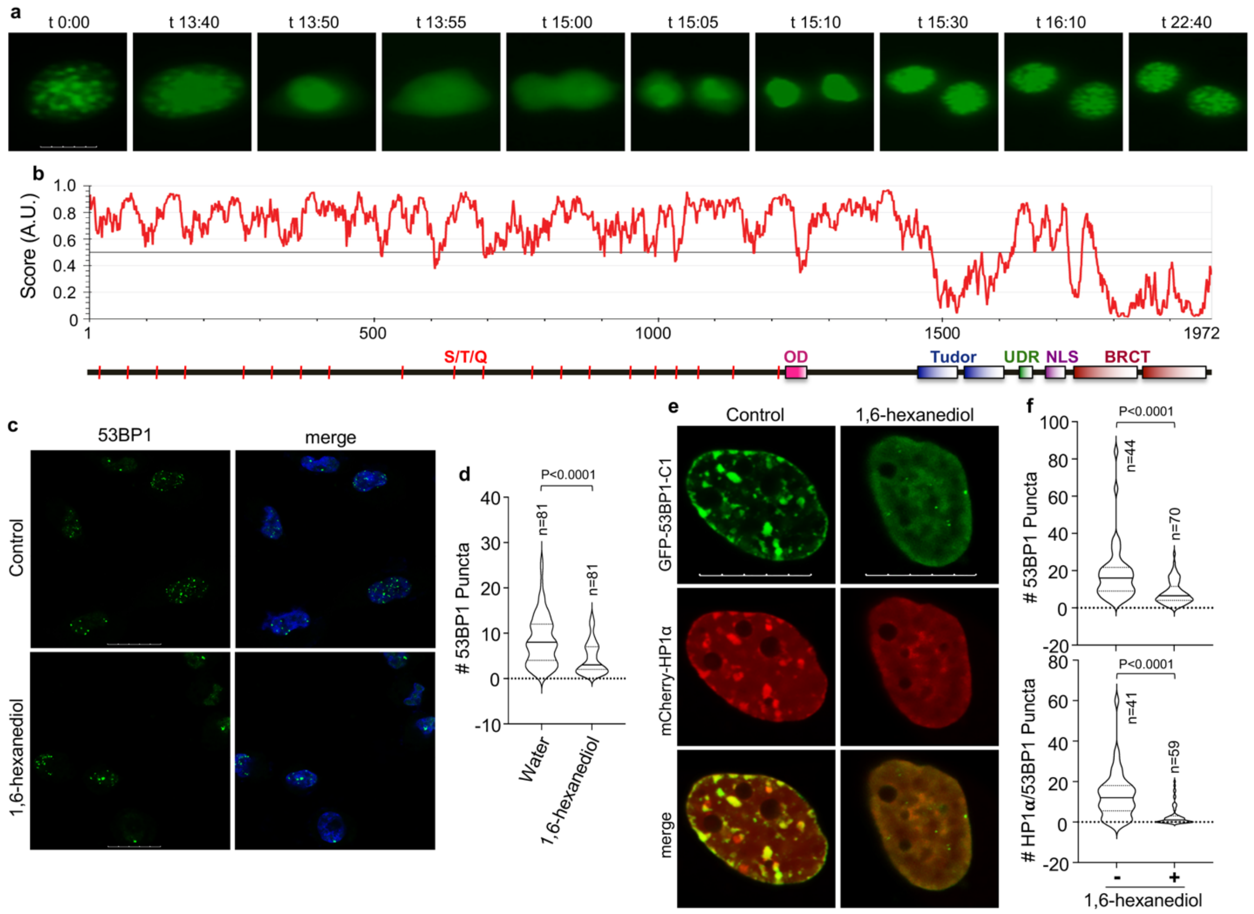

Supplementary Fig. 13. LLPS property of 53BP1 puncta. **a**, U-2 OS cells were transfected with GFP-53BP1-C1 for 24 h, and live cell imaging was conducted over time. Scale bar is 10 µm. **b**, IUPRED2A (https://iupred2a.elte.hu/) prediction of intrinsically disordered regions of human 53BP1. A score of 0 to 1 was assigned to each residue of 53BP1, indicating the probability of the given residue as being disordered. Higher values correspond to a higher probability of disorder. The domain structure of human 53BP1 is shown below. **c**, MDA-MB-231 parental cells grown on glass coverslips were treated with 5% 1,6-hexanediol for 5 min, fixed and stained with anti-53BP1 antibodies and visualized under confocal fluorescence microscopy. Representative single z-plane images are shown. Scale bar is 25 µm. **d**, Violin plot of 53BP1 puncta number per cell was analyzed from indicated numbers of cells in ***c*** done in two independent experiments. Data represent mean, 25th and 75th percentiles with the whiskers extending to the minimum and maximum values. **e**, U-2 OS cells were transfected with GFP-53BP1-C1 and mCherry-HP1α for 48 h and visualized under confocal fluorescence microscopy in the presence or absence of 5% 1,6-hexanediol for 10 min. Representative live cell single z-plane confocal images are shown. Scale bar is 25 µm. **f**, Violin plot of puncta number for GFP-53BP1-C1 (*upper*) and co-localized GFP-53BP1-C1/mCherry-HP1α (*lower*) was analyzed from indicated numbers of transfected cells. Data represent mean, 25th and 75th percentiles with the whiskers extending to the minimum and maximum values. Unpaired two-tailed t test using Prism 9.0 was performed in ***d*** and ***f*** with 95% confidence intervals.

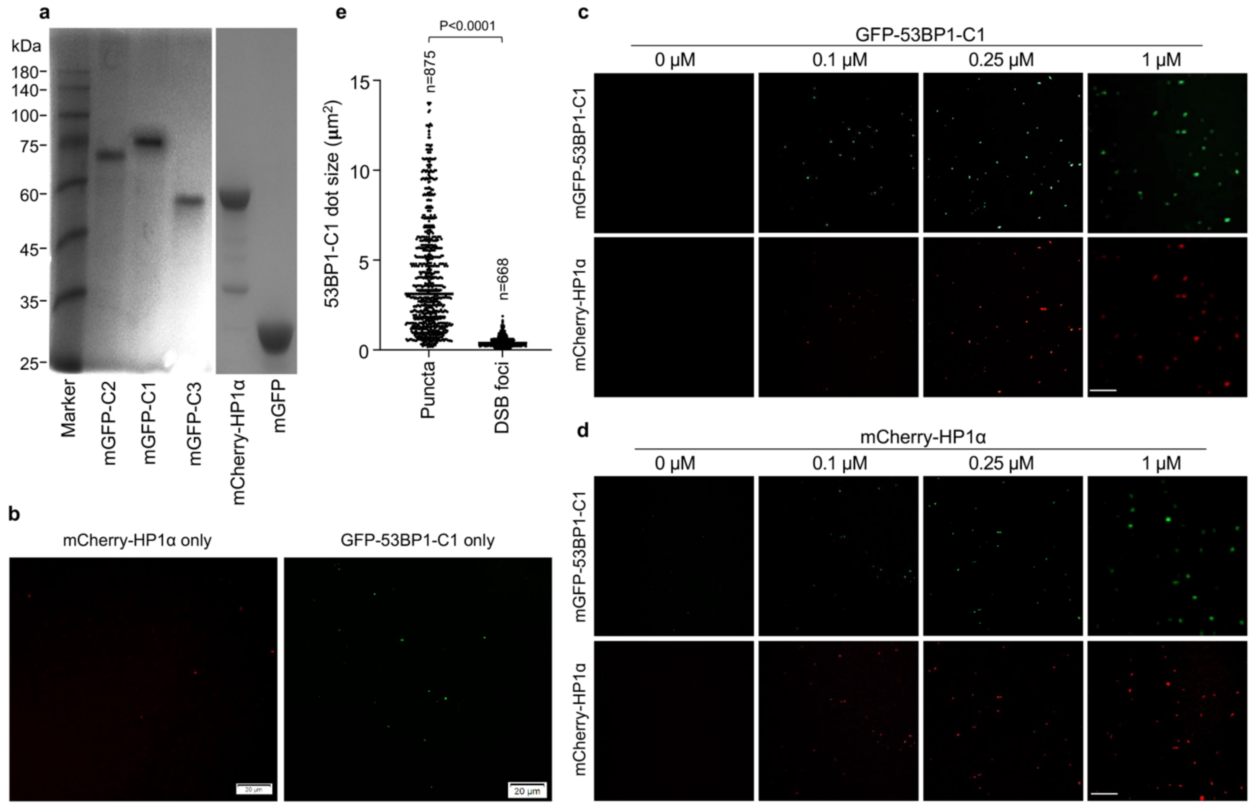

Supplementary Fig. 14. In vitro liquid droplet formation of 53BP1-C1 and HP1α. **a**, Coomassie staining of purified proteins. **b**, Representative single z-plane confocal images of purified GFP-53BP1-C1 or mCherry-HP1α alone in vitro at a concentration of 10 μM. **c**, Purified mCherry-HP1α proteins (10 μM) were mixed with increasing concentrations of GFP-53BP1-C1 and visualized under confocal microscopy. Representative single z-plane images were shown. **d**, Purified GFP-53BP1-C1 proteins (10 μM) were mixed with increasing concentrations of mCherry-HP1α and visualized under confocal microscopy. Representative single z-plane images were shown. Scale bar is 50 μm in ***c*** and ***d***. **e**, U-2 OS cells were transfected with GFP-53BP1-C1 for 48 h, treated with 3.5 μM bleomycin for 6 h, fixed and stained with anti-γH2AX. GFP-53BP1-C1 puncta and DSB foci were determined by their co-localization with γH2AX. Size distribution of GFP-53BP1-C1 puncta or DSB foci from n=875 and 668 events, respectively, acquired from two independent experiments was analyzed by the NIH Image J software. The median size of puncta and DSB foci was 3.120 and 0.377 μm^2 (in area), respectively. The 25%, 75% and 95% percentiles for GFP-53BP1-C21 puncta and DSB foci were 1.499 vs 0.274, 5.706 vs 0.592, and 10.360 vs 1.037 μm^2, respectively. Unpaired two-tailed t test using Prism 9.0 was performed for statistical analysis with 95% confidence intervals.

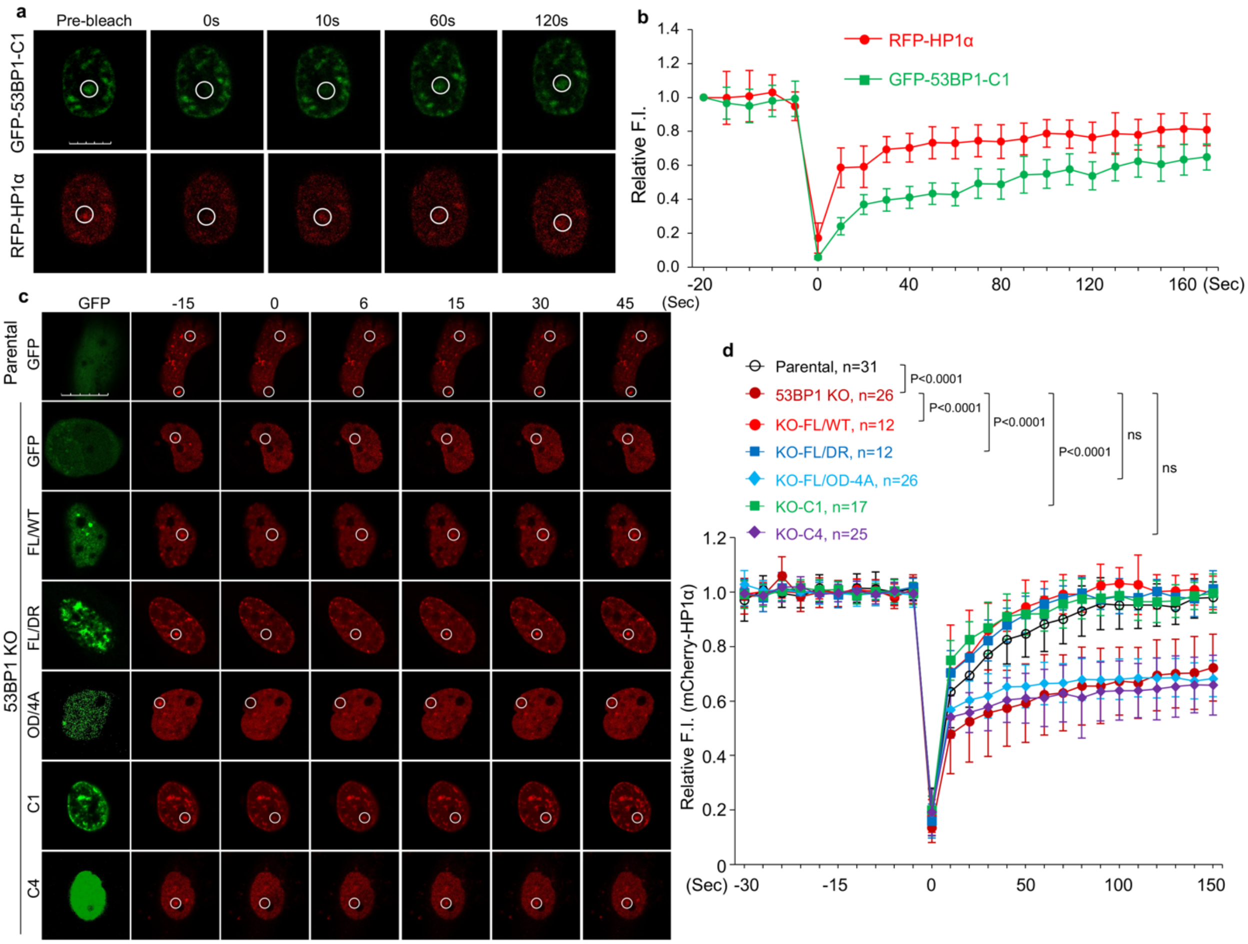

Supplementary Fig. 15. FRAP of co-localized 53BP1-C1 and HP1α puncta. **a**, U-2 OS parental cells grown on glass coverslips were transfected with GFP-53BP1-C1 and mCherry-HP1α for 48 h and FRAP analysis was conducted. Representative single z-plane confocal images are shown. Circles indicate the punctum that was bleached. Scale bar is 10 µm. **b**, FRAP analysis from cells in ***a*** from duplicated experiments. Data represent mean values and SD from n=8 events. Time 0 indicates photobleaching. **c**, U-2 OS cells from parental, 53BP1 KO or KO but reconstituted with different GFP-53BP1 constructs grown on 35 mm dish with circled glass bottom were transfected with mCherry-HP1α for 48 h (GFP alone was also co-transfected for parental and KO cells only), and FRAP analysis was conducted for mCherry-HP1α. Representative images are shown. Circles indicate the puncta that were bleached. Scale bar is 10 µm. **d**, Quantitative FRAP analysis from ***c.*** Data represent mean values and SD from n=31, 26, 12, 12, 26, 17 and 25 events, respectively, acquired from two independent experiments. Unpaired two-tailed t test using Prism 9.0 was performed between indicated groups with 95% confidence intervals. Time 0 indicates photobleaching.

Fig. S1a

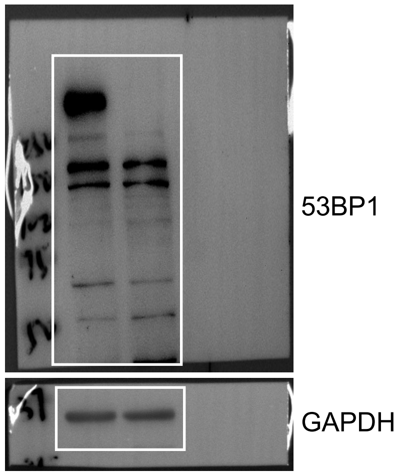

Fig. S1b

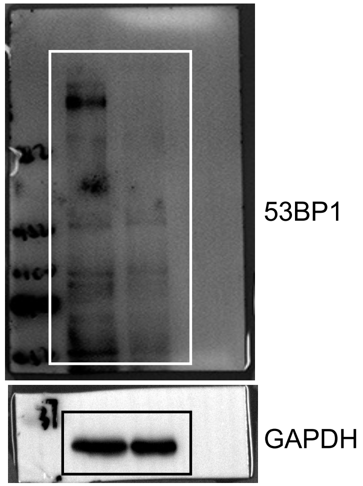

Fig. S1c

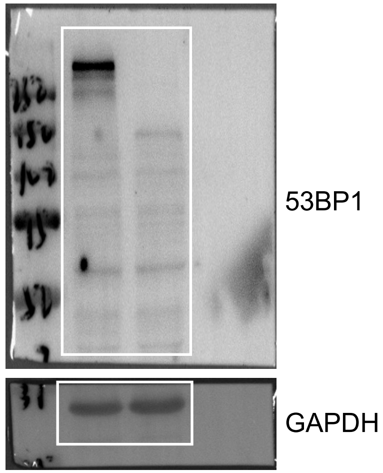

Fig. S1d

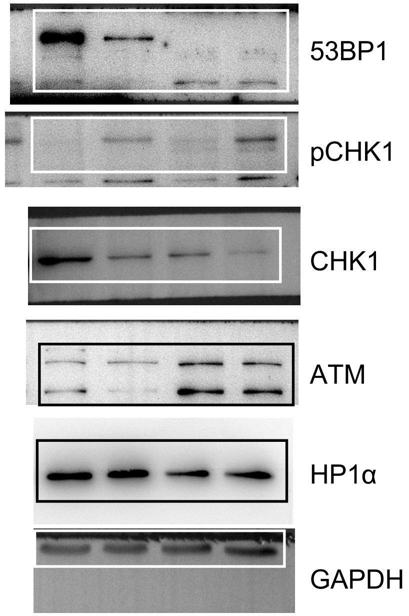

Fig. S1e

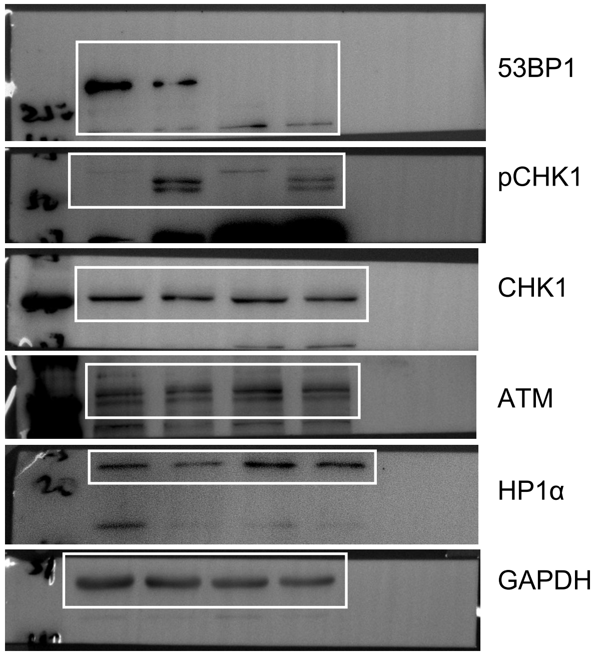

Fig. S1f

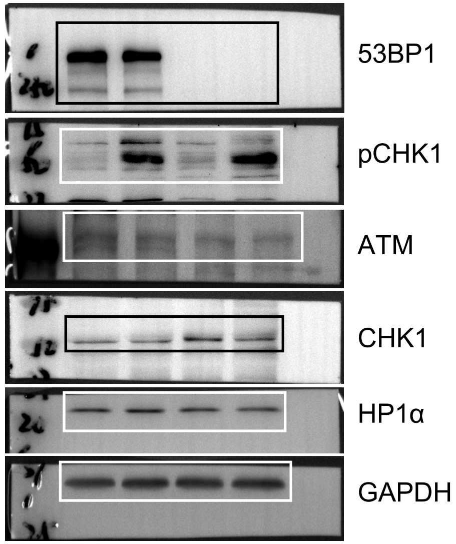

Fig. S10a

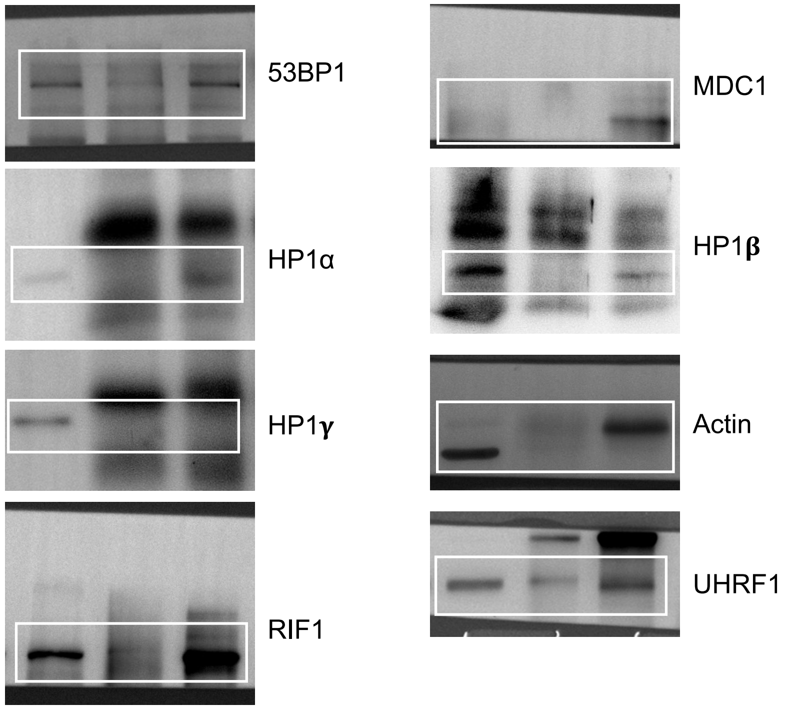

Fig. S10j

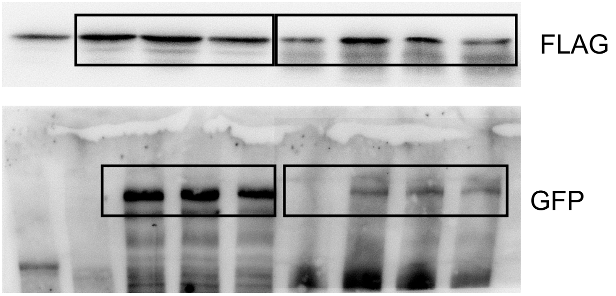

Fig. S10k

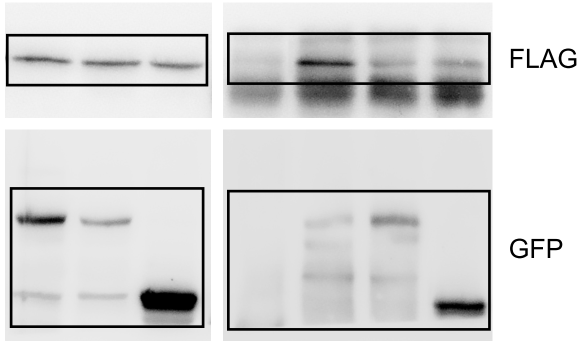

Supplement: Supplementary file 1 — Supplementary Information [file 41467_2022_28019_MOESM1_ESM.pdf]
