# Supplementary material for: 53BP1 regulates heterochromatin through liquid phase separation
Source: Nat Commun. 2022 Jan 18;13:360. doi: 10.1038/s41467-022-28019-y (PMC8766474; doi:10.1038/s41467-022-28019-y)
Supplement: Supplementary file 3 — Reporting Summary [file 41467_2022_28019_MOESM3_ESM.pdf]

nature research

Corresponding author(s): Youwei Zhang

Last updated by author(s): 12/14/2021

Reporting Summary

Nature Research wishes to improve the reproducibility of the work that we publish. This form provides structure for consistency and transparency in reporting. For further information on Nature Research policies, see our Editorial Policies and the Editorial Policy Checklist.

Statistics

For all statistical analyses, confirm that the following items are present in the figure legend, table legend, main text, or Methods section.

n/a	Confirmed	
☐	☒	The exact sample size (*n*) for each experimental group/condition, given as a discrete number and unit of measurement
☐	☒	A statement on whether measurements were taken from distinct samples or whether the same sample was measured repeatedly
☐	☒	The statistical test(s) used AND whether they are one- or two-sided<br>*Only common tests should be described solely by name; describe more complex techniques in the Methods section.*
☐	☒	A description of all covariates tested
☒	☐	A description of any assumptions or corrections, such as tests of normality and adjustment for multiple comparisons
☐	☒	A full description of the statistical parameters including central tendency (e.g. means) or other basic estimates (e.g. regression coefficient) AND variation (e.g. standard deviation) or associated estimates of uncertainty (e.g. confidence intervals)
☐	☒	For null hypothesis testing, the test statistic (e.g. *F*, *t*, *r*) with confidence intervals, effect sizes, degrees of freedom and *P* value noted<br>*Give P values as exact values whenever suitable.*
☒	☐	For Bayesian analysis, information on the choice of priors and Markov chain Monte Carlo settings
☒	☐	For hierarchical and complex designs, identification of the appropriate level for tests and full reporting of outcomes
☐	☒	Estimates of effect sizes (e.g. Cohen's *d*, Pearson's *r*), indicating how they were calculated

Our web collection on statistics for biologists contains articles on many of the points above.

Software and code

Policy information about availability of computer code

Data collection	Live cell imaging was collected by the Leica DMI6000 system with adaptive focus. Confocal and FRAP was acquired through the Leica TCS SP8 Hyvolution confocal microscopy system with a 63 and 40X oil objective lens, respectively, used for optimum resolution for bleaching with the Lasos LGK 7872 ML05 laser and the FRAP software LAS X version.
Data analysis	Live cell images were acquired every 5 min for 24-48 h with the appropriate settings for GFP and phase contrast. For FRAP, the size and depth of points-of-interest was determined in snapshots of cells under ideal optical parameters. Fluorescence images were analyzed using Image J software (v1.52q). Comet assay was analyzed by the Casp Lab software (v1.2.3b2). Photoshop (v21.2.1 and 23.0.2). ChIP-seq data were downloaded from ENCODE (https://www.encodeproject.org/), NCBI (https://www.ncbi.nlm.nih.gov/gds), EMBL-EBL ArrayExpress database (https://www.ebi.ac.uk/arrayexpress/experiments/E-MTAB-5817/) and SRA (https://www.ncbi.nlm.nih.gov/sra/?term=SRR10540101). Other softwares used include Trim Galore v0.6.5 (http://www.bioinformatics.babraham.ac.uk/projects/trim_galore/), Picard v1.114 (http://broadinstitute.github.io/picard), Pearson's Correlation calculation (https://www.socscistatistics.com/tests/pearson/default2.aspx) and IUPRED2A (https://iupred2a.elte.hu/).

For manuscripts utilizing custom algorithms or software that are central to the research but not yet described in published literature, software must be made available to editors and reviewers. We strongly encourage code deposition in a community repository (e.g. GitHub). See the Nature Research guidelines for submitting code & software for further information.

Data

Policy information about availability of data

All manuscripts must include a data availability statement. This statement should provide the following information, where applicable:

- Accession codes, unique identifiers, or web links for publicly available datasets
- A list of figures that have associated raw data
- A description of any restrictions on data availability

ChIP-seq data for 53BP1 were downloaded from GEO database (https://www.ncbi.nlm.nih.gov/gds) (accession number: GSE108114) or E-MTAB-5817 from EMBL-EBL ArrayExpress database (https://www.ebi.ac.uk/arrayexpress/experiments/E-MTAB-5817/) accession numbers ERR2008219, ERR2720661, ERR2720666 and SRR10540101) and SRR10540101 from SRA (https://www.ncbi.nlm.nih.gov/sra/?term=SRR10540101). All sequencing reads were trimmed using Trim Galore v0.6.5 (http://www.bioinformatics.babraham.ac.uk/projects/trim_galore/). Reads were mapped to hg38 version of human reference genome using Bowtie2 v2.4.1 allowing only unique alignment. PCR duplicates were removed using Picard v1.114 (http://broadinstitute.github.io/picard). Peaks were called using MACS2 v2.1.1 with -p 0.05. Additional ChIP-seq data for H3K9me3 and H3K27ac were downloaded from ENCODE (https://www.encodeproject.org/).

Field-specific reporting

Please select the one below that is the best fit for your research. If you are not sure, read the appropriate sections before making your selection.

[x] Life sciences   [ ] Behavioural & social sciences   [ ] Ecological, evolutionary & environmental sciences

For a reference copy of the document with all sections, see nature.com/documents/nr-reporting-summary-flat.pdf

Life sciences study design

All studies must disclose on these points even when the disclosure is negative.

Sample size	No statistical method was used to pre-determine the sample size, because a sample size of at least 3 biological replicates was chosen based on previous experience and standards in the field, which also reached statistical significance. For immunofluorescence, sample sizes (n=cell number or analyzed images) are shown in each figure and described in the figure legend. For in vivo FRAP analysis, n represents individual puncta analyzed. For other analysis (e.g., ChIP-qPCR and qPCR), data represent mean values and standard deviation from at least three biological replicates.
Data exclusions	No data were excluded from the analysis.
Replication	53BP1 punctate fluorescence results were done multiple times by 6 individual investogators. ChIP-qPCR, qPCR, clonogenic survival and ELISA were acquired from at least three biological replicates. Comet assay was done twice with similar results and >50 cells were analyzed for each group. In vitro phase separation was done at least three times. In vivo FRAP was done twice and analyzed events range from n=14-24, which is sufficient to provide significant difference.
Randomization	Randomization does not apply to our analyses, because cell lines and/or treatment conditions were per-determined. Specifically, each cell line or treatment is specifically defined (overexpressing a particular protein or with a specific manipulation like gene depletion or agent treatment). Unlike animal studies, randomization was not applied to define sample groups.
Blinding	Blinding was not used in our analyses, because the groups of each experiment were pre-determinied and collected into distinct settings. Hence, we analyzed all collected samples based on their per-determined groups and each group has been clearly defined. Statistical analysis was provided to support our conclusion.

Reporting for specific materials, systems and methods

We require information from authors about some types of materials, experimental systems and methods used in many studies. Here, indicate whether each material, system or method listed is relevant to your study. If you are not sure if a list item applies to your research, read the appropriate section before selecting a response.

Materials & experimental systems

n/a	Involved in the study	
[ ]	[x]	Antibodies
[ ]	[x]	Eukaryotic cell lines
[x]	[ ]	Palaeontology and archaeology
[x]	[ ]	Animals and other organisms
[x]	[ ]	Human research participants
[x]	[ ]	Clinical data
[x]	[ ]	Dual use research of concern

Methods

n/a	Involved in the study	
[x]	[ ]	ChIP-seq
[x]	[ ]	Flow cytometry
[x]	[ ]	MRI-based neuroimaging

Antibodies

Antibodies used	Rat monoclonal anti-53BP1 (#933002, clone W17184B, lot #B295535mainly for IF) antibody was purchased from Biolegend (San Diego, CA, USA). Rabbit polyclonal anti-H3K9me3 (#07-442), anti-p-histone H2A.X (Ser139) (for staining, JBW301, # 05-636-MI), anti-p-HH3 (Ser10) (#06-570), anti-RAD51 (#PC-130), anti-Cyclin A (#C4710), anti-53BP1 (#MAB3802), anti-CS35 (SC-35, #S4045) and anti-H2AK15ub (#MABE1119) antibodies were from Millipore/Sigma. Rabbit monoclonal anti-HA (C29F4, #3724S), anti-HP1a (#2616S), anti-HP1b (#8676S), and anti-SUV39H1 (#8729) antibodies were from Cell Signaling Technology (Danvers, MA, USA). Mouse anti-HP1α (GA-62, #SC-130446), anti-β-Actin Antibody (C4, #SC-47778) and anti-HP1γ (sc-398562) were from Santa Cruz Biotechnology (Santa Cruz, CA, USA). Anti-53BP1 (#NB100-304 and #NBP2-25028), anti-RIF1 (for staining, # NB100-1587), anti-TRF2 (#NB100-56506) and anti-GFP (#NB100-1770) antibodies were from Novus Biologicals (Centennial, CO, USA). Anti-ATM (2C1, #GTX70103) was from GeneTex Inc. (Irvine, CA, USA). Anti-pS824-KAP1 (#ab70369) was purchased from Abcam (Cambridge, UK).<br><br>Alexa Fluor conjugated secondary antibodies were purchased from Thermo/Invitrogen as follows. Chicken anti-mouse IgG (H+L) Alexa Fluor 647 (#A-214463), Chicken anti-rabbit IgG (H+L) Alexa Fluor 647 (#A-21443), Donkey anti-mouse IgG (H+L) Alexa Fluor 568 (#A-10037), Donkey anti-rabbit IgG (H+L) Alexa Fluor 568 (#A-10042), Donkey anti-mouse IgG (H+L) Alexa Fluor 488 (#A-21202), Donkey anti-rabbit IgG (H+L) Alexa Fluor 488 (#A-21206), Donkey anti-rat IgG (H+L) Alexa Fluor 488 (#A-21208), Chicken anti-rat IgG (H+L) Alexa Fluor 488 (#A-21470).
Validation	We only used those antibodies that have been validated previously by us or by the vendors. These include common tag antibodies, immunofluorescence antibodies and ChIP antibodies. Our knockout or knockdown experiments performed in this study (for instance, Fig 1-3, 5 and 7, and Supplementary Fig. 1-5, 8-10, 13 and 15, validate the antibodies for 53BP1, HP1s, GFP and FLAG.

Eukaryotic cell lines

Policy information about cell lines

Cell line source(s)	Regular U2OS, MCF10A, MDA-MB-231, HEK293T and IMR-90 cell lines were from ATCC. MCF10A 53BP1 KO cells were provided by Dr. Zihua Gong (CCF); U2OS and MDA-MB-231 53BP1 KO cells were provided by Dr. Kuntian Luo in Dr. Zhunkun Lou's lab (Mayo Clinic); MEF parental and 53BP1 KO cells were from Dr. Neil Johnson (Fox Chase Cancer Center) by isolating from 12.5-13.5 days postcoitum embryos. ARPE-19 cells were provided from Dr. Beata Jastrzebsak (CWRU), which was originally from ATCC.
Authentication	Cell lines have been authenticated. The recent authentication was done in May 2020 using the standard Promega STR assay. The authenticity of these cell lines including the acquired MCF10A cell line was confirmed.
Mycoplasma contamination	Mycoplasma contamination has been routinely tested. All cell lines were negative. Nonetheless, we have routinely treated cells with anti-mycoplasma agents (Plasmocin from InvivoGen).
Commonly misidentified lines<br>(See ICLAC register)	no commonly misidentified lines were used in this study
