# Supplementary figures and images for: 53BP1 regulates heterochromatin through liquid phase separation
Source: Nat Commun. 2022 Jan 18;13:360. doi: 10.1038/s41467-022-28019-y (PMC8766474; doi:10.1038/s41467-022-28019-y)

Fig. 1a

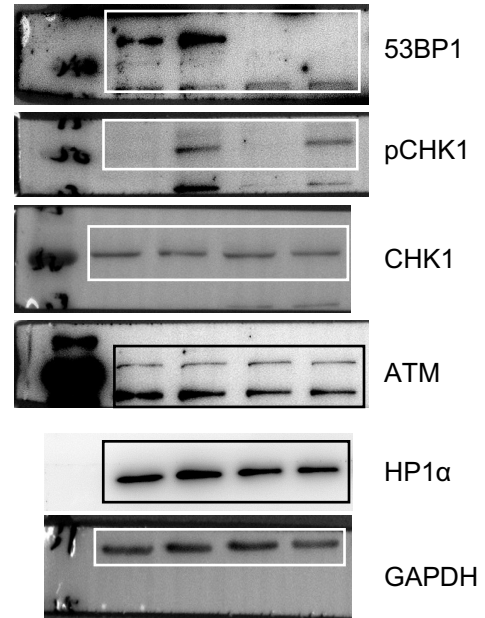

Fig. 3a

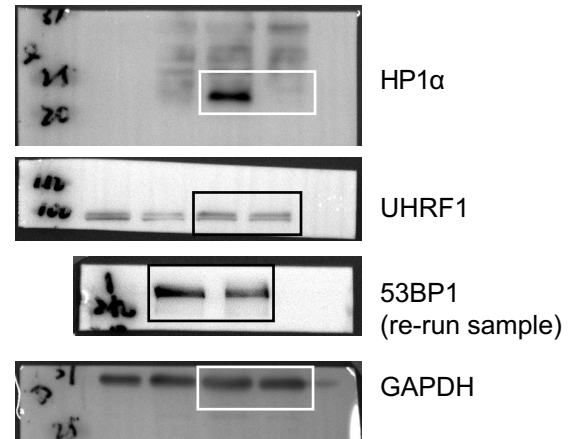

Fig. 3d

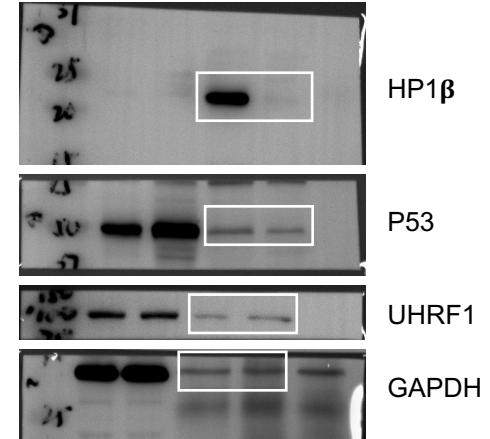

Fig. 5d

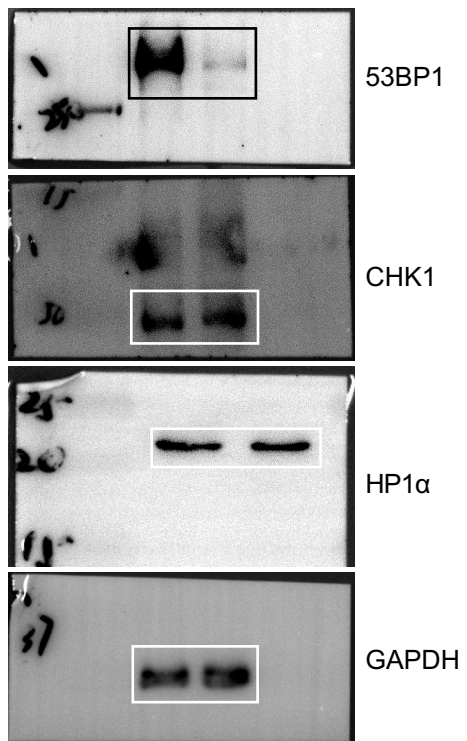

Fig. 5f

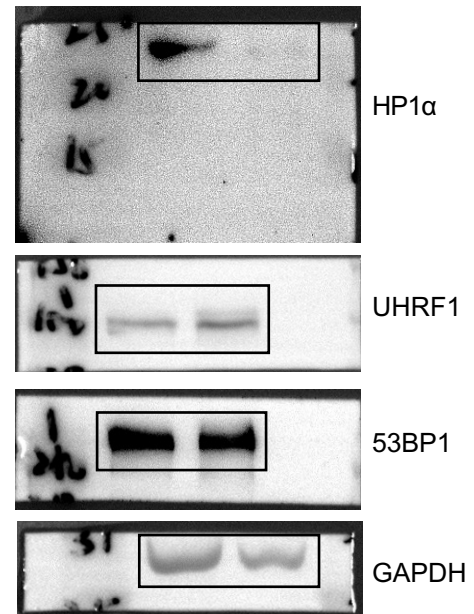

Supplement: Supplementary file 4 — Source data [file 41467_2022_28019_MOESM4_ESM.zip › Source Data/327731_2_data_set_6140466_r44nqw.pdf]
